# Revealing Hidden Patterns: Harnessing Correspondence Analysis to Explore Maternal Lifestyle Profiles and Gestational Weight Gain

**DOI:** 10.3390/nu18183038

**Published:** 2026-09-17

**Authors:** Mulubirhan Assefa Alemayohu, Federica Loperfido, Lucia Cazzoletti, Maria Elisabetta Zanolin, Dana El Masri, Irene Bianco, Chiara Ferrara, Rosa Maria Cerbo, Stefano Ghirardello, Beatrice Maccarini, Francesca Sottotetti, Francesca Garofoli, Micol Angelini, Maria Cristina Monti, Hellas Cena, Rachele De Giuseppe

**Affiliations:** 1 Biostatistics and Clinical Epidemiology Unit, Department of Public Health, Experimental and Forensic Medicine, University of Pavia, 27100 Pavia, Italy; 2 Laboratory of Dietetics and Clinical Nutrition, Department of Public Health, Experimental and Forensic Medicine, University of Pavia, 27100 Pavia, Italy; federica.loperfido@unipv.it (F.L.); dana.elmasri01@universitadipavia.it (D.E.M.); irene.bianco@unipv.it (I.B.); chiara.ferrara01@unipv.it (C.F.); beatrice.maccarini01@universitadipavia.it (B.M.); hellas.cena@unipv.it (H.C.); rachele.degiuseppe@unipv.it (R.D.G.); 3 Unit of Epidemiology & Medical Statistics, University of Verona, 37134 Verona, Italy; lucia.cazzoletti@univr.it (L.C.); elisabetta.zanolin@univr.it (M.E.Z.); 4 Neonatal Unit and Neonatal Intensive Care Unit, Fondazione IRCCS Policlinico San Matteo, 27100 Pavia, Italy; rm.cerbo@smatteo.pv.it (R.M.C.); s.ghirardello@smatteo.pv.it (S.G.); lab.immunoneo@smatteo.pv.it (F.G.); micol090777@gmail.com (M.A.); 5 Clinical Nutrition Unit, Department of General Medicine, Istituti Clinici Scientifici Maugeri IRCCS, 27100 Pavia, Italy

**Keywords:** pregnancy, maternal health, weight gain, gestational, correspondence analysis

## Abstract

**Background/Objectives**: Inadequate gestational weight gain (GWG), encompassing both low and excessive, is a significant concern because of its association with adverse maternal and neonatal health outcomes. The relationships between maternal lifestyle factors, such as diet, physical activity, prepregnancy body mass index (BMI), and GWG, are complex. This study aims to explore these associations via correspondence analysis (CA), a method that visualizes the relationships between categorical variables. **Methods:** A CA was conducted using baseline data from the ongoing LIMIT (Lifestyle and Microbiome Interaction Early Adiposity Rebound in Children) prospective cohort study. The analysis included 162 subjects whose complete maternal characteristic data were available to explore associations between maternal features and GWG. **Results**: The results were visualized through a two-dimensional graphical representation, with the related statistical measures. The horizontal axis of the CA map captures 63.4% of the total inertia of the data, and the vertical axis captures 36.6%. Maternal profiles with lower education, smoking, high meat consumption, and prepregnancy overweight or obesity showed a higher prevalence of excess GWG than did the average profile. Compared with the average profile, medium Mediterranean diet adherence was associated with a greater prevalence of low GWG and underweight and obese mothers with adequate GWG. **Conclusions:** This study identifies maternal lifestyle factors, such as diet and physical activity, as key determinants of GWG, with excess GWG linked to unhealthy behaviors. Tailored interventions focusing on prepregnancy BMI and dietary habits could optimize pregnancy outcomes. Personalized prenatal care is essential for managing GWG and improving maternal health.

## 1. Introduction

The global prevalence of inadequate gestational weight gain (GWG) is a significant concern because of its association with adverse maternal and neonatal health outcomes, including increased risks of preterm birth, low birth weight, and compromised maternal health [1]. Studies have shown that inadequate GWG is associated with adverse mental health outcomes [1], as demonstrated by a prospective cohort study conducted at Policlinico Abano (Abano Terme, Italy), which revealed that inadequate GWG is a warning sign of symptoms of anhedonia, anxiety, and depression [2]. A meta-analysis of studies from 25 countries revealed that the prevalence of insufficient GWG was 54%, whereas the prevalence of excessive weight gain was 22% [3]. A regional surveillance system in Italy revealed that 2306 (36.0%) out of 6405 women were within the appropriate weight gain range at delivery, whereas 2021 (31.6%) had insufficient weight gain and 2073 (32.4%) had excessive weight gain [4].

Excessive GWG is of particular clinical concern because it has been associated with adverse pregnancy outcomes, including gestational diabetes mellitus, gestational hypertension, pre-eclampsia, cesarean delivery, and fetal macrosomia [5,6]. Maternal diet represents an important modifiable factor influencing GWG. Higher energy intake and dietary patterns characterized by energy-dense and ultra-processed foods, often rich in fats, saturated fats, refined carbohydrates, and added sugars, have been associated with greater or excessive GWG [7], whereas healthier dietary patterns characterized by higher consumption of fruits, vegetables, nuts, legumes, and other minimally processed foods have generally been associated with more favorable GWG [8].

Beyond overall dietary patterns, adequate maternal intake of specific nutrients, including folate, iron, iodine, vitamin D, choline, and long-chain omega-3 fatty acids such as docosahexaenoic acid (DHA), is particularly important during pregnancy [9]. These nutrients contribute to key processes including maternal hematological and metabolic adaptation, placental function, neural tube formation, thyroid-dependent neurodevelopment, and fetal brain and skeletal development [9,10].

Understanding the mediating role of gestational weight gain in the relationship between maternal status and adverse birth outcomes is crucial [11]. Furthermore, a comprehensive understanding of how various maternal lifestyle factors, such as individual, family, and social influences [12], physical activity [13,14], and dietary habits [15], are associated with gestational weight gain is needed to explore their relationships. A higher prepregnancy BMI (classified as overweight or obese) [16], poor nutritional choices [17], lack of physical activity [14], and higher levels of education [3] were also found to be associated with an increased risk of excessive GWG. Adherence to the Mediterranean diet [18], regular physical activity [14], and normal prepregnancy BMI [19] were found to be associated with optimal GWG.

The relationships between maternal lifestyle factors and GWG are complex and multifaceted. Traditional analytical methods often struggle to capture the multidimensional and categorical nature of these relationships. CA [20] is a powerful tool uniquely suited to uncovering hidden patterns within datasets, as it visualizes the categories of categorical variables in an intuitive two-dimensional space. This ability makes CA [21] particularly valuable for exploring relationships among multiple categorical variables. Its strength lies in its ability to graphically represent associations, offering clear and intuitive insights. For example, CA can reveal how specific combinations of maternal lifestyle behaviors are linked with deviations in GWG categories, such as excessive or low weight gain, both of which have significant implications for maternal and fetal health. By uncovering these relationships, CA offers actionable insights that can enhance the development of tailored interventions. The goal of integrating CA with data from the LIMIT study is to provide a comprehensive overview of maternal behavior profiles and their correspondence with GWG categories, generating insights to guide future interventions aimed at promoting healthy behaviors in women planning pregnancies.

This study, therefore, aimed to use CA to identify and characterize maternal lifestyle profiles in relation to GWG categories among pregnant women who participated in the baseline of the ongoing Lifestyle and Microbiome Interaction Early Adiposity Rebound in Children (LIMIT) prospective cohort study [22].

## 2. Materials and Methods

### 2.1. Overview

LIMIT [22] is a prospective cohort study aimed at identifying the longitudinal interaction between the infant’s gut microbiome and the lifestyle and environmental variables of the infant/mother, focusing on children with early adiposity-bound (EAR) versus adiposity-rebound (AR). The study planned to enroll 272 mother–infant pairs and collect data at six time points: at the time of delivery (T0) and at one month (T1), six months (T2), 12 months (T3), 24 months (T4) and 36 months (T5) after birth. Women were recruited during pre-hospital care before delivery, whereas the baseline assessment, T0, corresponded to delivery. Final eligibility related to neonatal status, including term birth and absence of protocol-defined neonatal complications, was therefore confirmed at delivery. The present analysis was based on baseline LIMIT data collected at T0.

### 2.2. Study Design and Setting

A correspondence analysis was conducted using baseline data (T0) from 162 subjects, with no missing maternal characteristic data, who were enrolled during prehospital care before birth at the UOC Neonatology and Neonatal Intensive Care Unit, Fondazione IRCCS Policlinico San Matteo of Pavia. Participants were selected according to the inclusion criteria established in the study protocol [22]: having healthy infants at term, parents willing to provide informed consent and complete questionnaires, and having either elective cesarean section or vaginal delivery. Healthy term newborns were defined as infants born between 37 and 42 completed weeks of gestation who did not meet the protocol-defined neonatal exclusion criteria.

CA offers substantial potential for investigating the complex interplay between GWG and maternal lifestyle during pregnancy. Unlike traditional statistical analyses, which typically focus on linear relationships and isolated variables, CA excels at uncovering patterns and associations within categorical data. It is particularly effective for examining relationships among multiple categorical variables, offering intuitive insights through graphical representations of these associations.

### 2.3. Data Sources and Management

The studied variables and collection timing have been described in the study protocol [22], including maternal data collected at delivery (T0) through validated questionnaires and structured interviews. The dataset encompasses sociodemographic information (such as age and education level), prepregnancy anthropometric measurements (weight and height), calculated prepregnancy body mass index (BMI), and lifestyle behaviors during pregnancy (including eating habits, adherence to the Mediterranean diet, physical activity levels, and smoking habits). Further details on the variables studied and their measurements can be found in previously published articles [22,23]. Specifically, the adherence to the Mediterranean diet and the physical activity scores were constructed from sets of eight and five questions, respectively, whereas GWG was calculated as the difference between weight at the time of delivery and prepregnancy weight and was then classified into three categories on the basis of the guidelines issued by the Institute of Medicine (IOM) and the prepregnancy BMI categories. A summary of the dataset characteristics included in this study is provided in Table 1.

### 2.4. Statistical Analysis

A matrix was created to organize and structure the data, with maternal characteristics as rows and GWG outcomes as columns. A contingency table was then produced to summarize the frequency counts for each combination of row and column categories, resulting in 13 stacked cross-tables of each categorical maternal characteristic and GWG weight gain, with 162 observations for each cross-table. Subsequently, CA was employed to explore and visualize the relationships between categorical variables, with the graphical representation interpreted via key statistical measures, including inertia, eigenvalues, contributions, and factorial coordinates. The contribution of rows (profiles) or columns (outcome categories) to the dimension illustrates the variability in the data explained by the dimensions [20], highlighting the relationships among the categories of maternal features in the sample. Inertia is a measure of spread; in other words, it is a measure of the weighted spread of the profile row points describing the maternal characteristics according to the distribution of GWG. The map is the solution of CA and describes the spread of the profile row points around the average profile. We draw an asymmetric map to properly interpret the distance between profile row points (maternal features) and column vertices (GWG categories) as proxies for association.

## 3. Results

### 3.1. Characteristics of the Analytical Sample

The correspondence analysis included 162 women with complete data for all selected maternal characteristics. Among them, 63 (38.9%) had adequate gestational weight gain, 37 (22.8%) had excess gestational weight gain, and 62 (38.3%) had low gestational weight gain. The distributions of the sociodemographic, anthropometric, dietary, and lifestyle characteristics included in the analysis are presented in Table 1.

### 3.2. Overall Correspondence Analysis Solution

**Figure 1:**
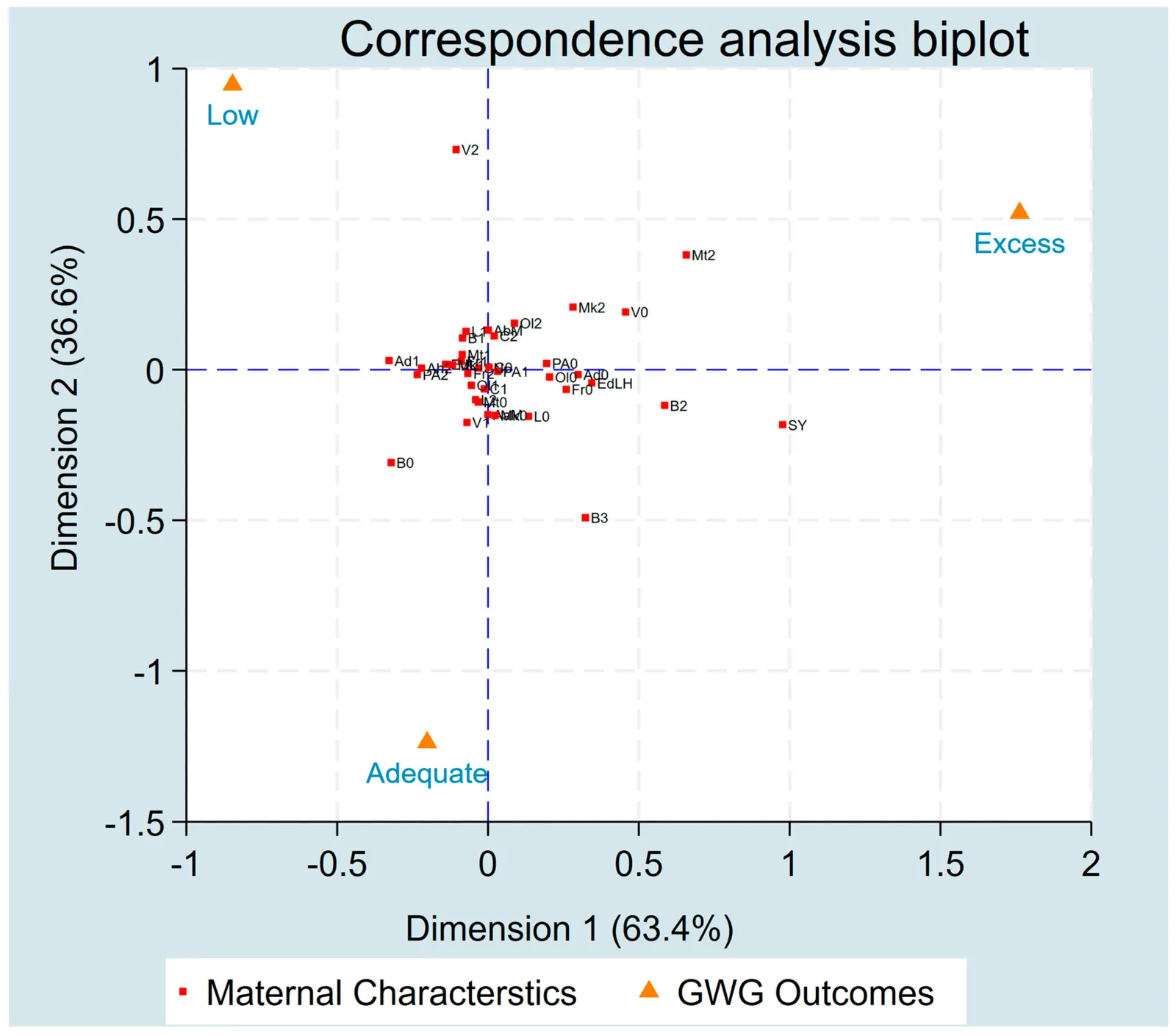
Correspondence plot for profiling maternal lifestyle by Gestational Weight Gain outcome [AbM—Below Mean Age, AaM—Above Mean Age, EdLH—High School or lower, EdU—University degree, SY—Smoker during pregnancy, SN—No smoking during pregnancy, B0—Underweight, B1—Normal, B2—Overweight, B3—Obesity, PA0—Lower Physical Activity score, PA1—Medium Physical Activity score, PA2—Higher Physical Activity score, Ad0—Lower Adherence to MD Score, Ad1—Medium Adherence to MD Score, Ad2—Higher Adherence to MD Score, Fr0—Inadequate daily fruit consumption, Fr1—Average daily fruit consumption, Fr2—Adequate daily fruit consumption, V0—Inadequate daily vegetable consumption, V1—Average daily vegetable consumption, V2—Adequate daily vegetable consumption, L0—Inadequate weekly legume consumption, L1—Average weekly legume consumption, L2—Adequate weekly legume consumption, C0—Inadequate daily cereal consumption, C1—Average daily cereal consumption].

Table 2 summarizes the relative frequency of adequate, low, and excess GWG for the categories of selected maternal characteristics. The two axes of Figure 1 explain the total variability of the data, where the horizontal and vertical axes account for 63.4% and 36.6% of the total inertia, respectively. Table 3 details the mass, inertia and contributions of each row and column category that determines the overall dimensional structure of the CA.

In Dimension 1, excess GWG accounts for 71.0% of the inertia, suggesting that this category contributes more significantly to the underlying patterns captured in this dimension. A low GWG contributes 27.4%, showing a moderate contribution, whereas an adequate GWG contributes only 1.6%, reflecting a minimal role in determining the inertia in Dimension 1. In contrast, Dimension 2 is influenced by an adequate GWG, which accounts for 59.5% of the inertia, highlighting its importance in the interpretation of this dimension. Low GWG also plays a significant role, contributing 34.3%, whereas excess GWG makes a minimal contribution of only 6.2% (Table 3).

### 3.3. Interpretation of Dimensions 1 and 2

Dimension 1 reflects weight-related behaviors and eating patterns associated with excessive weight or unhealthy lifestyles (such as being overweight, high meat consumption, and smoking). The most influential row profile in Dimension 1 is overweight, which contributes to 11% of the inertia explained by this dimension. This is closely followed by poor adherence to the Mediterranean diet (MD), which contributes 10.7%, indicating a similar level of influence. The consumption of more than two servings of meat and having a high school education or lower contributes to 9% and 8.9%, respectively. A lower consumption of vegetables per day and being a smoker contribute to 7.7% and 7.6%, respectively, reflecting a slightly lower but still notable influence in determining the spread around the average profile along Dimension 1 (Table 3). Table 2 reported that women reporting higher adequate vegetable consumption were more prevalent in the low GWG group (68.18%) than in either the adequate (4.55%) or excess (27.27%) groups, whereas higher daily meat consumption (>1.5 portions) was more common in the excess GWG group (53.85%). Smoking during pregnancy was strongly associated with excess GWG (60%), whereas nonsmoking was more evenly distributed among the Adequate and Low GWG categories. Figure 1 represents the CA map. According to the results of the analysis, specific row category profile points, such as inadequate vegetable consumption, high meat intake, and smoking, are plotted near the Excess GWG points. Profile row points representing higher adherence to the Mediterranean diet and a high intake of fruit and vegetables were generally closer to the Adequate or Low GWG.

Dimension 2 emphasized vegetable consumption, with adequate daily consumption of vegetables dominating the explanation of the variance in this dimension, as it represented 32.6% of the variance explained by this dimension. This is followed by average daily consumption of vegetables, which contributes 10%, while being affected by obesity plays a minor role, accounting for only 6.7% of the inertia in Dimension 2 (Table 3).

### 3.4. Correspondence Between Maternal Profiles and GWG Categories

According to the row percentages reported in Table 2, mothers with a high school or lower level of education (EdLH), who smoke during pregnancy (SY), consume more than 1.5 portions of meat (Mt2) and milk (Mk2) daily, have an inadequate daily consumption of vegetables (V0) and fruit (Fr0), have lower physical activity (PA0), have lower Mediterranean diet adherence scores (Ad0), and are affected by overweight (B2) or obesity (B3) before pregnancy presented a higher prevalence of excess GWG with respect to the average. Considering the distance between the corresponding profile row points and the points standing for the three categories of GWG in the CA map, the considered profile points are located closer to the Excess GWG point.

The profile row points representing mothers with a high school or lower educational level (EdLH), particularly those who are prepregnancy overweight (B2) or obese (B3), appear to be positioned in opposite directions along the first dimension relative to the center of the graph with respect to low GWG. This finding indicates a negative association between these maternal profiles and low GWG. On the other hand, mothers with a medium Mediterranean diet adherence score (Ad1) seem to have a slight positive association with low GWG. Additionally, mothers who are underweight or affected by obesity appear to have a higher prevalence of adequate GWG than the average profile does. All of the profile row points located near the origin of the CA map represent categories of women with a distribution of GWG similar to the average.

## 4. Discussion

The findings from this study provide insight into the complex relationship between maternal characteristics and GWG, with the CA dimensions capturing distinct influences of maternal lifestyle characteristics on GWG. Maternal profiles with lower education, smoking, high meat consumption, and prepregnancy overweight or obesity were associated with excess GWG. Compared with the average profile, medium Mediterranean diet adherence was slightly associated with low GWG; underweight and again obese mothers were more frequently associated with adequate GWG.

Most studies affirm that excessive GWG is strongly influenced by maternal lifestyle factors, including diet, physical activity, and sociodemographic elements such as education level and smoking status [24]. In our study, Dimension 1 was more involved in describing lifestyle factors associated with excessive GWG. Specifically, mothers with lower educational levels, as well as those who smoke during pregnancy and those who consume higher amounts of meat and dairy products, are at a greater risk of experiencing excess GWG. Furthermore, mothers with inadequate consumption of vegetables and fruits, low physical activity, and prepregnancy overweight status also have a greater prevalence of excessive GWG. The substantial contributions of these factors highlight a pattern associated with an increased likelihood of excess GWG, thereby emphasizing the need for tailored interventions aimed at reducing excessive weight gain during pregnancy by promoting healthier dietary habits, particularly among mothers showing these characteristics. For example, a Cochrane review by Muktabhant and colleagues [25] evaluated the effectiveness of diet and exercise interventions and concluded that these strategies could reduce the risk of excessive GWG by an average of 20%. Furthermore, an evidence report from the US Preventive Services Task Force highlights the effectiveness of lifestyle interventions, including dietary counseling, in achieving recommended levels of GWG [26].

Beyond its implications for maternal health, inadequate GW—both below and above recommended ranges—may also influence fetal development and contribute to long-term offspring health through fetal programming [27]. In fact, according to the Developmental Origins of Health and Disease (DOHaD) framework [28], the intrauterine nutritional and metabolic environment can induce developmental adaptations that affect the structure and function of organs and metabolic pathways throughout life. Insufficient maternal weight gain may limit fetal nutrient availability and is associated with impaired fetal growth, whereas excessive GWG may expose the fetus to an environment characterized by increased availability of glucose and lipids, insulin resistance, inflammation, and oxidative stress [27]. These conditions may affect placental nutrient transport and endocrine signaling and contribute to alterations in fetal adipogenesis, insulin signaling, appetite regulation, and energy metabolism [27]. Epigenetic mechanisms, including changes in DNA methylation, histone modifications, and non-coding RNA expression, have been proposed as potential mediators linking maternal nutritional status and GWG to persistent changes in offspring gene expression and subsequent susceptibility to obesity and cardiometabolic disorders [27]. Thus, achieving GWG within recommended ranges may be relevant not only for reducing adverse pregnancy outcomes, but also for promoting a more favorable intrauterine environment and long-term metabolic health of the offspring.

While a prior study by Hirko and colleagues [29] reported that higher vegetable intake was associated with lower odds of excessive GWG among women with obesity, our CA reveals a more nuanced picture. In our study, we also found that vegetable consumption is a key contributor to the variability captured in Dimension 2, indicating its importance in distinguishing GWG categories. However, unlike previous findings, those with adequate daily vegetable consumption in our sample did not consistently show adequate GWG, and in fact, their GWG was more often classified as low or excessive. This could suggest that while vegetable intake is an important dietary factor, other lifestyle or physiological elements (such as prepregnancy BMI) might affect GWG. Meat consumption, in addition to vegetable consumption, lies farther from the center and thus has a stronger relationship with how GWG outcomes diverge. These findings highlight the complexity of dietary patterns during pregnancy, suggesting that no single food group alone can guarantee adequate GWG without consideration of overall nutritional balance and other lifestyle factors. Interestingly, these findings are also highlighted in studies [30,31] suggesting that individualized interventions focused on a healthy lifestyle, diet, and exercise can improve behaviors and increase the proportion of women achieving GWG within the Institute of Medicine (IOM) recommendations.

According to some studies, women who are overweight or obese are more likely to experience excessive gestational weight gain (EGWG) than their normal-weight counterparts [32,33]. However, the U.S. Preventive Services Task Force (USPSTF) reported that behavioral counseling interventions aimed at healthy weight management are particularly valuable for women with elevated prepregnancy BMIs, as these interventions can lead to more favorable GWG patterns [34]. Our analysis reveals that the row profile points standing for mothers affected by prepregnancy underweight (B0) and obesity (B3) are nearer to adequate GWG than to inadequate (excess or low) GWG, showing that these groups tend to have a higher prevalence of adequate GWG than the average profile. This finding could suggest that baseline body composition might influence the likelihood of achieving adequate GWG, serving as a proxy for counseling that significantly affects how dietary and physical activity interventions are tailored during pregnancy. Furthermore, our analysis revealed that prepregnancy weight status could moderate GWG, with mothers in higher BMI categories being less likely to experience low GWG. According to USPSTF’s recommendation statement [34], the use of prepregnancy BMI as a factor in dietary counseling can significantly increase the effectiveness of prenatal care by addressing individual nutritional needs and promoting healthier pregnancy outcomes.

The relevance of appropriate GWG may also extend to breastfeeding outcomes. For instance, excessive GWG has been associated with a higher risk of delayed lactogenesis II and shorter exclusive breastfeeding duration [35]. In fact, a recent systematic review and meta-analysis reported a 28% higher risk of delayed lactogenesis II among women with excessive GWG [35], while previous evidence suggests that both inadequate and excessive GWG may negatively affect breastfeeding initiation and duration, partly depending on prepregnancy BMI [36]. Possible mechanisms include insulin resistance, inflammation, altered hormonal responses, and obstetric complications that may interfere with lactation [36]. Evidence on the relationship between GWG and human milk quantity and nutritional quality is less consistent. Maternal nutritional and metabolic status may influence milk production and the concentration of selected nutrients and bioactive compounds, while overweight and obesity have been associated with changes in milk lipid and hormonal composition [37]. However, the independent contribution of GWG remains difficult to distinguish from that of prepregnancy BMI and maternal diet [37].

This study leverages CA to show clusters and trends in maternal lifestyle behaviors, easing a nuanced understanding of the factors contributing to variations in GWG. Furthermore, the method adds significant value to the design of educational pathways that promote proper lifestyle choices among women planning a pregnancy. By pinpointing the most influential lifestyle factors and their interconnections, CA enables a targeted approach to intervention strategies, such as personalized educational programs for women at greater risk of inadequate GWG. This study, which employs CA, presents strengths and limitations in examining the relationship between maternal characteristics and GWG.

The strengths of this type of analysis are the effective visualization of complex relationships, the dimension reduction for easier interpretation, and the ability to identify significant lifestyle patterns. On the other hand, CA only allows a description of the relationships among the studied variables and is unable to show causation. Other limitations of the study are the use of a questionnaire developed for healthy women, small sample size effects, the exclusion of alcohol-related data, assumptions in CA on linear relationships that may oversimplify interactions, and the inability to establish causation. Moreover. some maternal exposures, including prepregnancy weight and lifestyle behaviors during pregnancy, were retrospectively reported at delivery and may therefore be subject to recall or reporting bias. Recognizing these factors is essential for interpreting findings and developing targeted maternal health interventions.

## 5. Conclusions

In conclusion, this study reveals noteworthy variations in maternal profiles associated with adequate (AGWG), excessive (EGWG), and low (LGWG) GWG outcomes. The findings show that mothers affected by being underweight or obese are more likely to achieve AGWG, highlighting the potential benefits of early nutritional counseling. Targeted interventions focusing on lifestyle factors and prepregnancy BMI are essential for improving maternal health and improving pregnancy outcomes.

## Figures and Tables

**Table 1:** Distribution of maternal characteristics and gestational weight gain categories in the complete-case sample included in the correspondence analysis (n = 162).

Characteristics	Frequency (%)
**GWG adequacy**	
Adequate GWG	63 (38.9%)
Excess GWG	37 (22.8%)
Low GWG	62 (38.3%)
**Age Group**	
Below mean age (≤33 years)	86 (53.1%)
Above mean age (>33 years)	76 (46.9%)
**Mother’s Educational Level**	
Lower or High School	47 (29.0%)
University degree	115 (71.0%)
**Did you smoke during pregnancy?**	
NO	157 (96.9%)
YES	5 (3.1%)
**Prepregnancy BMI**	
Underweight	16 (9.9%)
Normal weight	116 (71.6%)
Overweight	20 (12.3%)
Obesity	10 (6.2%)
**Physical Activity**	
Lower Score	55 (34.0%)
Medium Score	54 (33.3%)
Higher Score	53 (32.7%)
**Mediterranean diet Adherence Score category**	
Lower Score	75 (46.3%)
Medium Score	30 (18.5%)
Higher Score	57 (35.2%)
**Daily Fruits Consumption**	
Inadequate	40 (24.7%)
Average	102 (63.0%)
Adequate	20 (12.3%)
**Daily Vegetables Consumption**	
Inadequate	23 (14.2%)
Average	117 (72.2%)
Adequate	22 (13.6%)
**Daily CEREAL consumption**	
Inadequate	26 (16.0%)
Average	88 (54.3%)
Adequate	48 (29.6%)
**Weekly Fish Consumption**	
Inadequate	66 (40.7%)
Average	89 (54.9%)
Adequate	7 (4.3%)
**Daily Meat Consumption**	
Less than 1 portion	79 (48.8%)
1 to 1.5 portion	70 (43.2%)
>1.5 portion	13 (8.0%)
**Daily milk and milk product Consumption**	
Less than 1 portion	50 (30.9%)
1 to 1.5 portion	82 (50.6%)
>1.5 portion	30 (18.5%)
**Olive Oil Consumption**	
Occasional	13 (8.0%)
Regular	110 (67.9%)
Frequent	39 (24.1%)

**Table 2:** Maternal lifestyle profile by gestational weight gain categories.

Maternal Characteristics	GWG Adequacy, n = 162		
	Adequate (%)	Excess (%)	Low (%)
Age Group			
Below Mean Age	32.56	24.42	43.02
Above Mean Age	46.05	21.05	32.89
Educational Level			
High School or Lower	38.30	36.17	25.53
University Degree	39.13	17.39	43.48
Smoking during Pregnancy			
No	38.85	21.66	39.49
Yes	40.00	60.00	0.00
Physical Activity Score			
Lower	36.36	30.91	32.73
Medium	38.89	24.07	37.04
Higher	41.51	13.21	45.28
Adherence to MD Score			
Lower	37.33	34.67	28.00
Medium	40.00	10.00	50.00
Higher	40.35	14.04	45.61
Prepregnancy BMI			
Underweight	56.25	6.25	37.50
Normal	34.48	20.69	44.83
Overweight	40.00	45.00	15.00
Obesity	60.00	30.00	10.00
Daily Fruit Consumption			
Inadequate	40.00	32.50	27.50
Average	38.24	19.61	42.16
Adequate	40.00	20.00	40.00
Daily Vegetable Consumption			
Inadequate	26.09	43.48	30.43
Average	47.86	17.95	34.19
Adequate	4.55	27.27	68.18
Weekly Legume Consumption			
Inadequate	45.28	26.42	28.30
Average	33.33	21.43	45.24
Adequate	44.00	20.00	36.00
Daily Cereal Consumption			
Inadequate	38.46	23.08	38.46
Average	42.05	21.59	36.36
Adequate	33.33	25.00	41.67
Daily Meat Product Consumption			
Less than 1 portion	44.30	20.25	35.44
1 to 1.5 portion	37.14	20.00	42.86
>1.5 portion	15.38	53.85	30.77
Daily Milk and Milk Product Consumption			
Less than 1 portion	46.00	22.00	32.00
1 to 1.5 portion	39.02	18.29	42.68
>1.5 portion	26.67	36.67	36.67
Average Profile	38.89	22.84	38.27

**Table 3:** Summary of contributions, mass, and inertia for row and column categories.

Maternal Characteristics	Overall		Contributions	
	Mass	%Inert	Dimension 1	Dimension 2
**Row Categories**				
Age Group				
Below Mean Age	0.041	0.015	0	0.041
Above Mean Age	0.036	0.017	0	0.047
Educational Level				
High School or Lower	0.022	0.057	0.089	0.003
University Degree	0.055	0.023	0.036	0.001
Smoking during Pregnancy				
No	0.075	0.002	0.002	0
Yes	0.002	0.05	0.076	0.005
Prepregnancy BMI				
Underweight	0.008	0.032	0.026	0.042
Normal	0.055	0.021	0.013	0.036
Overweight	0.009	0.072	0.11	0.008
Obesity	0.005	0.035	0.017	0.067
Physical Activity Score				
Lower	0.026	0.021	0.033	0.001
Medium	0.026	0.001	0.001	0
Higher	0.025	0.03	0.047	0
Adherence to MD Score				
Lower	0.036	0.068	0.107	0.001
Medium	0.014	0.033	0.052	0.001
Higher	0.027	0.028	0.044	0
Daily Fruit Consumption				
Inadequate	0.019	0.029	0.043	0.005
Average	0.048	0.009	0.013	0.002
Adequate	0.009	0.001	0.001	0
Daily Vegetable Consumption				
Inadequate	0.011	0.057	0.077	0.023
Average	0.056	0.042	0.009	0.1
Adequate	0.01	0.122	0.004	0.326
Weekly Legume Consumption				
Inadequate	0.025	0.023	0.015	0.035
Average	0.04	0.018	0.007	0.038
Adequate	0.012	0.003	0.001	0.007
Daily Cereal Consumption				
Inadequate	0.012	0	0	0
Average	0.042	0.004	0	0.01
Adequate	0.023	0.006	0	0.017
Daily Meat Product Consumption				
Less than 1 portion	0.038	0.01	0.001	0.025
1 to 1.5 portion	0.033	0.007	0.008	0.005
>1.5 portion	0.006	0.076	0.09	0.052
Daily milk and milk product Consumption				
Less than 1 portion	0.024	0.012	0	0.032
1 to 1.5 portion	0.039	0.012	0.018	0.001
>1.5 portion	0.014	0.037	0.038	0.036
**Column Categories**				
Gestational Weight Gain (GWG) Adequacy				
Adequate GWG	0.389	0.228	0.016	0.595
Excess GWG	0.228	0.473	0.71	0.062
Low GWG	0.383	0.299	0.274	0.343

## Data Availability

The datasets used and/or analyzed during the current study are available from the corresponding author on reasonable requests with permission of participating organizations.

## References

[1] Martinez-Hortelano J.A., Cavero-Redondo I., Alvarez-Bueno C., Garrido-Miguel M., Soriano-Cano A., Martinez-Vizcaino V. (2020). Monitoring gestational weight gain and prepregnancy BMI using the 2009 IOM guidelines in the global population: A systematic review and meta-analysis. BMC Pregnancy Childbirth.

[2] Zanardo V., Giliberti L., Giliberti E., Grassi A., Perin V., Parotto M., Soldera G., Straface G. (2021). The role of gestational weight gain disorders in symptoms of maternal postpartum depression. Int. J. Gynaecol. Obstet..

[3] Darling A.M., Wang D., Perumal N., Liu E., Wang M., Ahmed T., Christian P., Dewey K.G., Kac G., Kennedy S.H. (2023). Risk factors for inadequate and excessive gestational weight gain in 25 low- and middle-income countries: An individual-level participant meta-analysis. PLoS Med..

[4] Pani P., Carletti C., Giangreco M., Knowles A., Clagnan E., Gobbato M., Del Zotto S., Cattaneo A., Ronfani L., Gestational Weight Survey G. (2023). Monitoring gestational weight gain: Setting up a regional surveillance system in Italy. BMC Public Health.

[5] Voerman E., Santos S., Inskip H., Amiano P., Barros H., Charles M.A., Chatzi L., Chrousos G.P., Corpeleijn E., LifeCycle Project-Maternal Obesity and Childhood Outcomes Study Group (2019). Association of Gestational Weight Gain with Adverse Maternal and Infant Outcomes. JAMA.

[6] Ren M., Li H., Cai W., Niu X., Ji W., Zhang Z., Niu J., Zhou X., Li Y. (2018). Excessive gestational weight gain in accordance with the IOM criteria and the risk of hypertensive disorders of pregnancy: A meta-analysis. BMC Pregnancy Childbirth.

[7] Ferreira L.B., Lobo C.V., Miranda A., Carvalho B.D.C., Santos L.C.D. (2022). Dietary Patterns during Pregnancy and Gestational Weight Gain: A Systematic Review (Padroes alimentares durante a gravidez e ganho de peso gestacional: Uma revisao sistematica.). Rev. Bras. Ginecol. Obstet..

[8] Donovan S., Dewey K., Novotny R., Stang J., Taveras E., Kleinman R., Raghavan R., Nevins J., Scinto-Madonich S., Kim J.H. (2020). Dietary Patterns During Pregnancy and Gestational Weight Gain: A Systematic Review.

[9] Cetin I., Buhling K., Demir C., Kortam A., Prescott S.L., Yamashiro Y., Yarmolinskaya M., Koletzko B. (2019). Impact of Micronutrient Status during Pregnancy on Early Nutrition Programming. Ann. Nutr. Metab..

[10] De Giuseppe R., Roggi C., Cena H. (2014). n-3 LC-PUFA supplementation: Effects on infant and maternal outcomes. Eur. J. Nutr..

[11] Xiang X.M., Huang Y., Wang Z.P., Li Z.K., Dang S.N. (2024). Mediating role of gestational weight gain in the relationship between socioeconomic status and preterm birth: A Chinese population-based study. BMC Public Health.

[12] Samano R., Martinez-Rojano H., Ortiz-Hernandez L., Najera-Medina O., Chico-Barba G., Gamboa R., Mendoza-Flores M.E. (2023). Individual, Family, and Social Factors Associated with Gestational Weight Gain in Adolescents: A Scoping Review. Nutrients.

[13] Samano R., Martinez-Rojano H., Chico-Barba G., Gamboa R., Mendoza-Flores M.E., Robles-Alarcon F.J., Perez-Martinez I., Monroy-Munoz I.E. (2024). Gestational Weight Gain: Is the Role of Genetic Variants a Determinant? A Review. Int. J. Mol. Sci..

[14] Hamann V., Deruelle P., Enaux C., Deguen S., Kihal-Talantikite W. (2022). Physical activity and gestational weight gain: A systematic review of observational studies. BMC Public Health.

[15] Suliga E., Rokita W., Adamczyk-Gruszka O., Pazera G., Ciesla E., Gluszek S. (2018). Factors associated with gestational weight gain: A cross-sectional survey. BMC Pregnancy Childbirth.

[16] Kouba I., Del Pozzo J., Lesser M.L., Shahani D., Gulersen M., Bracero L.A., Blitz M.J. (2024). Socioeconomic and clinical factors associated with excessive gestational weight gain. Arch. Gynecol. Obstet..

[17] Zhou M., Peng X., Yi H., Tang S., You H. (2022). Determinants of excessive gestational weight gain: A systematic review and meta-analysis. Arch. Public Health.

[18] Radwan H., Hashim M., Hasan H., Abbas N., Obaid R.R.S., Al Ghazal H., Naja F. (2022). Adherence to the Mediterranean diet during pregnancy is associated with lower odds of excessive gestational weight gain and postpartum weight retention: Results of the Mother-Infant Study Cohort. Br. J. Nutr..

[19] Yang S., Peng A., Wei S., Wu J., Zhao J., Zhang Y., Wang J., Lu Y., Yu Y., Zhang B. (2015). Pre-Pregnancy Body Mass Index, Gestational Weight Gain, and Birth Weight: A Cohort Study in China. PLoS ONE.

[20] Greenacre M. (2016). Correspondence Analysis in Practice.

[21] Sourial N., Wolfson C., Zhu B., Quail J., Fletcher J., Karunananthan S., Bandeen-Roche K., Beland F., Bergman H. (2010). Correspondence analysis is a useful tool to uncover the relationships among categorical variables. J. Clin. Epidemiol..

[22] De Giuseppe R., Loperfido F., Cerbo R.M., Monti M.C., Civardi E., Garofoli F., Angelini M., Maccarini B., Sommella E., Campiglia P. (2022). LIMIT: LIfestyle and Microbiome InTeraction Early Adiposity Rebound in Children, a Study Protocol. Metabolites.

[23] El Masri D., Alemayohu M.A., Loperfido F., Bianco I., Ferrara C., Cerbo R.M., Ghirardello S., Monti M.C., Maccarini B., Sottotetti F. (2024). Associations of maternal lifestyle factors with inadequate pregnancy weight gain: Findings from the baseline data of the LIMIT prospective cohort study. Eur. J. Nutr..

[24] Harrison C.L., Bahri Khomami M., Enticott J., Thangaratinam S., Rogozinska E., Teede H.J. (2023). Key Components of Antenatal Lifestyle Interventions to Optimize Gestational Weight Gain: Secondary Analysis of a Systematic Review. JAMA Netw. Open.

[25] Muktabhant B., Lawrie T.A., Lumbiganon P., Laopaiboon M. (2015). Diet or exercise, or both, for preventing excessive weight gain in pregnancy. Cochrane Database Syst. Rev..

[26] Cantor A.G., Jungbauer R.M., McDonagh M., Blazina I., Marshall N.E., Weeks C., Fu R., LeBlanc E.S., Chou R. (2021). Counseling and Behavioral Interventions for Healthy Weight and Weight Gain in Pregnancy: Evidence Report and Systematic Review for the US Preventive Services Task Force. JAMA.

[27] Pawlikowska K., Koryszewska-Baginska A., Konieczna M., Oledzka G. (2026). Maternal Nutritional Status and Intrauterine Programming: Epigenetic Mechanisms and Their Impact on Offspring Health. Nutrients.

[28] Lacagnina S. (2020). The Developmental Origins of Health and Disease (DOHaD). Am. J. Lifestyle Med..

[29] Hirko K.A., Comstock S.S., Strakovsky R.S., Kerver J.M. (2020). Diet during Pregnancy and Gestational Weight Gain in a Michigan Pregnancy Cohort. Curr. Dev. Nutr..

[30] Menichini D., Spelta E., Monari F., Petrella E., Facchinetti F., Neri I. (2024). Lifestyle Intervention to Promote an Adequate Gestational Weight Gain and Improve Perinatal Outcomes in a Cohort of Obese Women. Nutrients.

[31] Aşcı Ö.S., Rathfisch G. (2016). Effect of lifestyle interventions of pregnant women on their dietary habits, lifestyle behaviors, and weight gain: A randomized controlled trial. J. Health Popul. Nutr..

[32] Deputy N.P., Sharma A.J., Kim S.Y., Hinkle S.N. (2015). Prevalence and Characteristics Associated With Gestational Weight Gain Adequacy. Obstet. Gynecol..

[33] Viteri O.A., Whitty J.E., Salazar X.C., Refuerzo J. (2015). Maternal and Infant Implications of Excessive Gestational Weight Gain among Obese Pregnant Women. J. Endocrinol. Diabetes Obes..

[34] Force U.S.P.S.T., Davidson K.W., Barry M.J., Mangione C.M., Cabana M., Caughey A.B., Davis E.M., Donahue K.E., Doubeni C.A., Krist A.H. (2021). Behavioral Counseling Interventions for Healthy Weight and Weight Gain in Pregnancy: US Preventive Services Task Force Recommendation Statement. JAMA.

[35] Cao Z., Huang M., Chen Y., Yin J., Li Y., Liu B., Peng J., Liu C.L. (2024). Association Between Gestational Weight Gain and Delayed Onset of Lactogenesis II: A Systematic Review and Meta-Analysis. Breastfeed. Med..

[36] Huang Y., Ouyang Y.Q., Redding S.R. (2019). Maternal Prepregnancy Body Mass Index, Gestational Weight Gain, and Cessation of Breastfeeding: A Systematic Review and Meta-Analysis. Breastfeed. Med..

[37] Ayande R., Sen S., Robinson D.T. (2026). Maternal diet and nutritional status during pregnancy and lactation: A review of implications on milk composition and lactation outcomes. Semin Perinatol..

